# Comparative Genomics of *Taphrina* Fungi Causing Varying Degrees of Tumorous Deformity in Plants

**DOI:** 10.1093/gbe/evu067

**Published:** 2014-03-28

**Authors:** Isheng J. Tsai, Eiji Tanaka, Hayato Masuya, Ryusei Tanaka, Yuuri Hirooka, Rikiya Endoh, Norio Sahashi, Taisei Kikuchi

**Affiliations:** ^1^Division of Parasitology, Faculty of Medicine, University of Miyazaki, Japan; ^2^Biodiversity Research Center, Academia Sinica, Taipei, Taiwan; ^3^Department of Environmental Science and Engineering, Ishikawa Prefectural University, Ishikawa, Japan; ^4^Forestry and Forest Products Research Institute, Tsukuba, Japan; ^5^Biodiversity (Mycology), Eastern Cereal and Oilseed Research Centre, Agriculture and Agri-Food Canada, Ottawa, Ontario, Canada; ^6^Microbe Division/Japan Collection of Microorganisms, RIKEN BioResource Center, Tsukuba, Japan

**Keywords:** *Taphrina* comparative genomics, divergence clusters, fungi aneuploidy

## Abstract

*Taphrina* fungi are biotrophic plant pathogens that cause plant deformity diseases. We sequenced the genomes of four *Taphrina* species—*Taphrina wiesneri*, *T. deformans, T. flavorubra*, and *T. populina*—which parasitize *Prunus*, *Cerasus,* and *Populus* hosts with varying severity of disease symptoms. High levels of gene synteny within *Taphrina* species were observed, and our comparative analysis further revealed that these fungi may utilize multiple strategies in coping with the host environment that are also found in some specialized dimorphic species. These include species-specific aneuploidy and clusters of highly diverged secreted proteins located at subtelomeres. We also identified species differences in plant hormone biosynthesis pathways, which may contribute to varying degree of disease symptoms. The genomes provide a rich resource for investigation into *Taphrina* biology and evolutionary studies across the basal ascomycetes clade.

## Introduction

Many different fungal pathogens induce deformities of host plants. One of the most well-known examples is the tumor-like plant structure caused by *Taphrina* species. The disease symptoms of deformity vary depending on the plant organ where infection occurs and which *Taphrina* species is the source of infection (Mix 1935). Infection symptoms include leaf spots, leaf curl, deformed fruits, and witches’ brooms. *Taphrina deformans,* which causes leaf curl of peach, is the most economically important species and has been extensively studied, and its genome sequence is now publicly available (Cissé et al. 2013). Another *Taphrina* species of interest is *T. wiesneri*, which causes serious damage to the culturally important “Sakura" (*Cerasus* spp.) in Japan. This fungus causes witches’ brooms on the twigs, loss of flowering, and early defoliation on *Cerasus* species. In particular, *Cerasus* × *yedoensis* cv. Somei-Yoshino, the most popular ornamental species in Japan, is highly susceptible to infection (Komatsu et al. 2010) and is endangered in some localities. The witches’ brooms is thought to be induced by plant hormones produced by systemically distributed perennial intercellular mycelium in twigs, hence the different disease symptoms induced by *Taphrina* species may be attributed to their ability to synthesize and modulate different plant hormones. In addition, *T. flavorubra* is the causal agent of deformed fruits or plum pocket on plums (Rodrigues and Fonseca 2003). Infected fruit increases abnormally in size, deforms, and becomes spongy. On the other hand, *T. populina* induces less deformity than others; this fungus infects *Populus* trees and causes only minor leaf spots (Rodrigues and Fonseca 2003).

The four *Taphrina* species are part of the order of Taphrinales, in the class Taphrinomycetes. Taphrinomycetes belong to the subphylum Taphrinomycotina, which includes the model fission yeast *Schizosaccharomyces pombe* and the human pathogen yeast-like fungus *Pneumocystis jirovecii.* Together they form a monophyletic group basal to the rest of ascomycetes (Liu et al. 2009). The genus *Taphrina* comprises about 28 species, which are distributed worldwide. Currently, all known *Taphrina* species are dimorphic: The mycelial (filamentous) state is strictly plant parasitic, whereas the yeast state is saprophytic and can be cultured on artificial media (Mix 1949; Rodrigues and Fonseca 2003). The yeast state of the life cycle begins with budding of ascospores discharged from infected leaf surfaces usually around late spring or early summer. These yeast-state cells make contact with host plants and switch to the parasitic mycelial state under favorable conditions. The parasitic phase is the predominant mode of the life cycle of these species, where the hyphae penetrate the cuticle and continue to invade the host tissue between the epidermal cells until they reach the parenchyma cells below (Mix 1935). Although all of the disease symptoms were thought to be attributed to the plant hormone-like substances such as indole-3-acetic acid (IAA) produced by *Taphrina* species, the exact mechanisms remain unclear. Two genes that are known to be required for IAA production for *Ustilago maydis* are also present in *T. deformans* (Cissé et al. 2013), suggesting that *T. deformans* and other *Taphrina* species might have a similar route of IAA biosynthesis.

Here, we report genome sequences of four major *Taphrina* species: *T. wiesneri*, *T. flavorubra*, *T. populina,* and a much-improved assembly of *T. deformans*. We have performed a comparative analysis with seven other fungal species and identified features unique to each *Taphrina* species that may be linked to their pathogenicity. This study provides valuable insights into the mechanisms underlying host specificity and different infection symptoms of *Taphrina* species.

## Materials and Methods

### Strain Collection and Condition

*Taphrina deformans* strain JCM 22205, *T. wiesneri* strain JCM 22204, *T. populina* strain JCM 22190, and *T. flavorubra* JCM 22207 were obtained from RIKEN BRC-JCM (Tsukuba, Japan). The isolation source of the strains used, host range, distribution, and disease symptom of the four *Taphrina* species are listed in supplementary table S1, Supplementary Material online.

### DNA/RNA Preparation and Sequencing

Yeast-like fungi were cultivated in YPD medium (0.5% (w/v) yeast extract, 1% peptone, and 0.9% glucose) at 25 °C for 3 days. The cells were collected from the medium by centrifugation (for 5 min at 3,000 × g) and were suspended in cell-wall lysis buffer (0.1 M McILvaine buffer containing 270 U/ml Westase (Takara Bio) and 0.5 M sodium tartrate). After incubation at 30 °C for 1.5 h, genomic DNA was extracted using Genomic-tip 100/G (Qiagen) according to manufacturer’s instruction. One microgram of the DNA was used to construct standard 300 bp libraries using the TruSeq DNA Sample Preparation Kit with the standard protocol (Illumina). Libraries were sequenced on an Illumina HiSeq2000 following the manufacturer’s recommended protocol to produce 100-bp paired end reads (https://icom.illumina.com/, last accessed April 4, 2014). RNA was extracted from yeast cells grown on YPD agar medium using Sepasol-RNA I Super (Nacalai Tesque) after cell disruption by tungsten beads (φ0.1 mm). RNA libraries were prepared with an Illumina TruSeq RNA Sample Prep Kit and sequenced (100-bp paired end) on an Illumina HiSeq2000 sequencer using the standard protocol (Illumina).

### Nuclear and Mitochondrial Genome Assembly

The initial assemblies of *Taphrina* genome sequences were produced using the MaSuRCA assembler (Zimin et al. 2013) from Illumina reads. These assemblies were further improved using the PAGIT approach (Swain et al. 2012). Gapfiller (Boetzer and Pirovano 2012) and IMAGE (Tsai et al. 2010) were used to close gaps and extend contig ends. The original Illumina reads were then remapped to the assemblies, and a separate assembly was produced with the unmapped Illumina reads using Velvet (Zerbino and Birney 2008) and further scaffolded into the main assemblies using SOPRA (Dayarian et al. 2010). Finally, iCORN v0.97 was run for three iterations to improve the consensus quality of the assemblies, and potential misassemblies were detected and broken using REAPR (Hunt et al. 2013). The CEGMA pipeline (Parra et al. 2007) was run to assess the genome completeness of these assemblies. The statistics of the final assemblies is shown in table 1.

**Table 1:** *Taphrina* Genome Assemblies

Species Name	Strain	Assembly Size (bp)	Number of Scaffolds	Average Scaff Size (kb)	Largest Scaff Size (kb)	N50 (kb)	N50 (Number)	N90 (kb)	N90 (Number)	Full Cegma (%)	GC Content (%)
*Taphrina wiesneri*	JCM 22204	13,070,656	225	58.1	584.8	304.1	17	71.6	47	98.79	47.8
*T. deformans*	JCM 22205 (= NRRL Y-17787)	13,778,166	529	26	398.7	182.6	28	25.9	97	98.79	48.9
*T. deformans*^a^	PYCC 5710 (= CBS 356.35)	13,364,053	394	33.9	244.1	71.9	61	17.5	203	98.7	49.5
*T. populina*	JCM 22190 (= CBS 337.55)	11,999,082	335	35.8	892.8	172.6	20	35.7	76	98.39	47.3
*T. flavorubra*	JCM 22207	15,727,932	865	18.2	480.9	177.6	30	7.9	149	98.39	48.9

^a^Published by Cissé et al. (2013).

Mitochondrial genome reads were assembled separately using MITObim (Hahn et al. 2013). Initial seeds were identified from the nuclear genome assemblies based on BLAST analyses using *S. pombe* mitochondrial genes as queries. Mitochondria assemblies were made by iterative mappings of the Illumina short reads to the initial seeds using kmers of 31 and 45. The MITOS web server (Bernt et al. 2013) was used for annotation of obtained assemblies.

### Genome Feature Analysis

Transposable elements (TEs) in the assembly of the four *Taphrina* species were identified based on output from two repeat detection programs RepeatModeler (http://www.repeatmasker.org/RepeatModeler.html, last accessed April 4, 2014) and TransposonPSI (http://transposonpsi.sourceforge.net/, last accessed April 4, 2014). UCLUST (Edgar 2010) was used to cluster these putative sequences (with 80% identity) to generate consensus sequences for a nonredundant library of repeat sequences. Annotations of these sequences were based on output of both programs and BLAST results from the National Center for Biotechnology Information (NCBI) nonredundant database. RepeatMasker (v3.2.8, http://www.repeatmasker.org, last accessed April 4, 2014) was used to calculate the distribution of each repeat and its abundance. Custom Perl scripts were used to choose the best match from overlapping matches in RepeatMasker output to avoid calculating the same region more than once. Illumina reads were remapped to the *Taphrina* genome assemblies using SMALT (v 0.7.5, available at http://www.sanger.ac.uk/resources/software/smalt/, last accessed April 4, 2014). The summary statistics of the read mappings is shown in supplementary table S9, Supplementary Material online). The GC content of 2-kb nonoverlapping windows in the assemblies was calculated using BEDtools (Quinlan and Hall 2010). Duplicates were called using GATK (McKenna et al. 2010), and median sequence coverage of 10-kb nonoverlapping window was calculated from the output of samtools (Li et al. 2009) and with a combination of custom perl and R scripts.

### Gene Prediction and Functional Annotations

RNAseq reads were aligned to the respective *Taphrina* genome assemblies using Tophat (v2.0.4 using parameters –mate-std-dev 50 -a 6 -i 10 -I 10000 –min-segment-intron 10 –max-segment-intron 10000; summary of the mappings is shown in supplementary table S9, Supplementary Material online) (Kim et al. 2013), and the mappings were converted into intron hints. Additionally, cufflinks (v2.0.1) (Trapnell et al. 2012) was used to assemble transcript fragments, and this information was converted into exon hints. Two additional sources of hints were available: The published *T. deformans* PYCC5710 proteome (Cissé et al. 2013) was transferred using RATT (Otto et al. 2011), and 2,863 *T. wiesneri* expressed sequence tag (EST) sequences were aligned using BLAT (Kent 2002). Initial gene models were first predicted based on these hints using Augustus (initial parameters were generated using the etraining script; Stanke et al. 2006). About 500 genes were curated for each *Taphrina* species using Artemis (Carver et al. 2008), and these models were used to train species-specific parameters in Augustus. The final sets of gene models were predicted based on trained parameters and concatenated hints in Augustus. Using *T. wiesneri* as an example, exon prediction sensitivity was increased from 76.6% to 84.6%, but specificity was decreased from 85.2% to 83.2% when comparing the repredicted gene models against the manual curated models. Signal peptides and transmembrane domains were detected using SignalP 4.0 (Petersen et al. 2011) and TMHMM 2.0 (Krogh et al. 2001), respectively. Codon usage and GC content of the codons’ third position was calculated using codonW (http://sourceforge.net/projects/codonw/, last accessed April 4, 2014). Functional annotations were predicted as means of GO terms by argot2 (Falda et al. 2012) and by identifying protein domains against Pfam v27.0 (Punta et al. 2012) using pfam_scan.pl (ftp://ftp.sanger.ac.uk/pub/databases/Pfam/Tools, last accessed April 4, 2014).

The MEROPS server was used to identify *Taphrina* putative peptidases. The peptidase candidates were derived from MEROPS batch BLAST (Rawlings et al. 2006). The candidates were manually examined in terms of similarity (*E*-value cutoff 1 × 10^−10^) to MEROPS proteins and presence of all catalytic sites. CAZymes (carbohydrate active enzymes) were detected using the dbCAN HMMer-based classification system (Yin et al. 2012) with an *E*-value threshold of 1 × 10^−5^, alignment length cutoff of 80 amino acids and covered fraction threshold of 0.3.

### Ortholog Clustering, Phylogeny, and Synteny Analysis

To construct a representative set of fungal gene families, nonredundant proteomes of *Saccharomyces cerevisiae*, *Sclerotinia sclerotiorum*, *Fusarium graminearum*, *Neurospora crassa, U. maydis,* and *P. jirovecii* were downloaded from Uniprot (version November 6, 2013), and *S. pombe* was downloaded from PomBase (Wood et al. 2012). Genes were clustered based on BLASTP (*E* value of 1 × 10^−5^) results and OrthoMCL (inflation parameter of 1.5, (Li et al. 2003). Amino acid alignment using MAFFT (Katoh and Standley 2013) of 75 single-copy orthologs with less than 10% alignment gaps were concatenated, and the distance matrix of the merged alignment was calculated using ProtTest (Abascal et al. 2005). The phylogenetic tree was constructed by maximum likelihood using RAxML (Stamatakis 2006) with 100 bootstrap replicates. From the OrthoMCL and Pfam output, we identified gene families that were lost and expanded in different positions along the global phylogeny leading to the *Taphrina* species. GO enrichments were carried out on these families using TopGO (version 2.10.0; Alexa and Rahnenfuhrer 2010).

The highly contiguated nature of the *Taphrina* spp. genome assemblies allowed for an analysis of scaffold synteny. To visualize synteny relationships between *Taphrina* species, dotplots of *T. wiesneri* versus the other three species were produced using the MUMmer 3.0 suite (Kurtz et al. 2004), and pairwise syntenic blocks were inferred using DAGchainer (Haas et al. 2004). To determine gene order rearrangements between *T. wiesneri* and *S. pombe,* we retrieved one-to-one orthologs between the two species from OrthoMCL output and visualized using circos (Krzywinski et al. 2009).

## Results and Discussion

## The Genomes and Gene Features of *Taphrina* Fungi

**Fig. 1.—:**
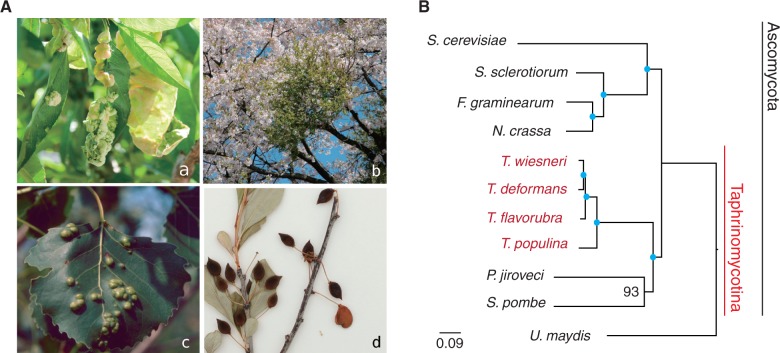
*Taphrina* infection symptoms and phylogeny. (*A*) Leaf curl (*a*), witches’ broom (*b*), leaf spots (*c*), and deformed fruits (*d*) that were caused by four different *Taphrina* species indicated by arrows. (*a*) Image no. 5435623: Yuan-Min Shen, Taichung District Agricultural Research and Extension Station, Bugwood.org; (*c*) image no. 3046084: Theodor D. Leininger, USDA Forest Service, Bugwood.org. (*B*) A maximum-likelihood phylogeny of four *Taphrina* species and seven other fungi from 75 single copy orthologs. Blue dots indicate 100% bootstrap support.

The nuclear genomes of *T. wiesneri, T. deformans, T. populina,* and *T. flavorubra* were selected for sequencing on the basis of the very different symptoms exhibited by their many *Prunus*, *Cerasus,* and *Populus* hosts worldwide (fig. 1*A*, supplementary table S1, Supplementary Material online). The *Taphrina* genomes were sequenced using Illumina technology with up to 450 × depth of coverage obtained. CEGMA analysis suggests that assemblies are 98.4–98.8% complete. The assemblies range from 12.0 to 15.7 Mb (table 1), similar to the 12.5-Mb genome of fission yeast *S. pombe*. Using transcriptomic evidence such as RNA Illumina sequenced reads and ESTs to guide de novo gene prediction, we identified 6,403–7,563 putative gene models per genome (table 2). Our *T. deformans* assembly is on average 99.7% identical with the published genome sequence of *T. deformans* PYCC5710 (Cissé et al. 2013) but is twice as contiguated. Similarly, the *Taphrina* mitochondrial genomes were assembled into single scaffolds ranging from 56 to 60 kb (supplementary fig. S1, Supplementary Material online), which is bigger than that reported for *S. pombe* (19.4 kb) (Bullerwell et al. 2003) but still smaller than *Sa. cerevisiae* (85.8 kb) (Foury et al. 1998). In the most contiguated case of *T. wiesneri*, the mitochondrial genome was assembled into a single sequence of 58,215 bp without any gaps, containing 39 genes (supplementary fig. S1*A*, Supplementary Material online), consisting of 14 proteins, 2 ribosomal RNAs, and 23 transfer RNAs. Our mitochondrial assembly of *T. deformans* is 56,006 bp in size, which is 20 kb bigger and identified 16 more tRNA genes than previously reported (Cissé et al. 2013). The gene orders of the four mitochondrial genomes are in almost perfect synteny to each other, with only minor differences in the order of tRNAs (supplementary fig. S1*B*, Supplementary Material online). Together these assemblies allow us to build upon what is already known about *Taphrina* species using a comparative genomics approach.

**Table 2:** *Taphrina* Gene Models Statistics

Species Name	Number of Genes	Gene Density (Genes/Mb)	Mean Exon per Gene	Mean Exon Length (bp)	Mean Intron Length (bp)
*Schizosaccharomyces pombe*	5,123	554	2.0	1,015	81
*Pneumocystis jirovecii*	3,898	481	3.7	211	61
*Taphrina wiesneri*	6,403	490	2.0	691	48
*T. deformans*	6,433	467	2.0	689	48
*T. populina*	6,481	540	2.0	656	51
*T. flavorubra*	7,563	481	1.9	704	50

The *Taphrina* genomes contain low numbers of TEs, with the largest class being LTR retrotransposons, which occupy about 1% of the genomes (supplementary table S2, Supplementary Material online). This observation partly explains that the highly contiguated nature of *Taphrina* assemblies, where 50% of the genomes are present in scaffolds at least 172 kb in length in spite of the fact that only short-insert Illumina libraries were used. A loss of TEs has occurred in *T. wiesneri* when compared with the others, where only 0.4% of the genome has signatures to any types of TEs.

## Phylogeny, Synteny, and Aneuploidy

**Fig. 2.—:**
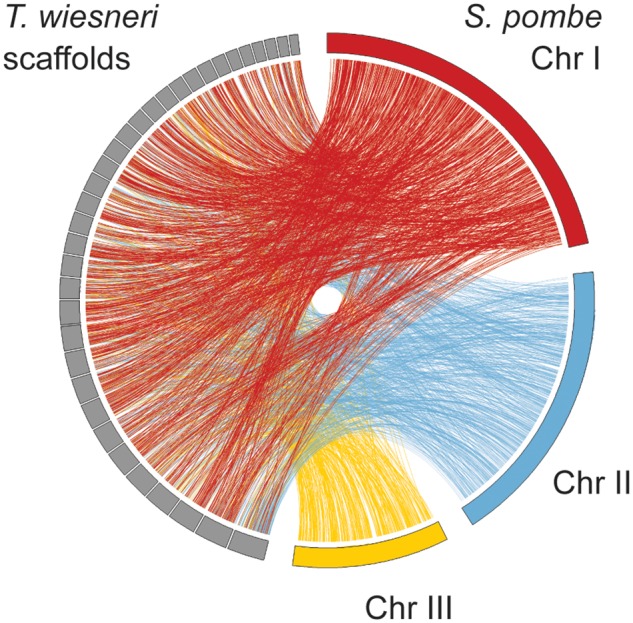
Lack of synteny between *Taphrina wiesneri* and *Schizosaccharomyces pombe.* One-to-one orthologs inferred from OrthoMCL (indicated by lines) connecting three *S. pombe* chromosomes against *T. wiesneri* scaffolds.

**Fig. 3.—:**
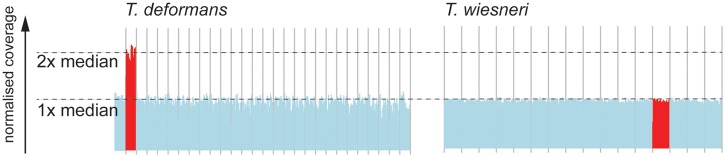
Distribution of normalized genome coverage on *Taphrina wiesneri* and *T. deformans.* Each vertical line indicates the median coverage of 100-kb windows normalized against the median coverage of either species. Even coverage was observed across the scaffolds of *T. wiesneri,* but in the case of *T. deformans,* the coverage was doubled across the second largest scaffold. Red color lines represent the coverage of such scaffold and the corresponding syntenic scaffold in *T. deformans*.

To identify gene gain and loss in *Taphrina* species, orthologous relationships were established using OrthoMCL (Li et al. 2003) against seven other fungi (fig. 1*B*). A phylogeny based on 75 single-copy orthologs placed the *Taphrina* species into a monophyletic clade relative to other Taphrinomycotina members, such as *S. pombe* and the human pathogen *P. jirovecii.* Together they form a clade of early diverging ascomycetes (fig. 1*B*). Unlike the fission yeasts (Rhind et al. 2011), the *Taphrina* genomes exhibit a high degree of synteny (supplementary fig. S2, Supplementary Material online). For the most closely related species of *T. wiesneri* and *T. deformans*, single-copy orthologs have on average 86.2% amino acid identity and show a strikingly high gene colinearity of 89%. Gene order and chromosomal synteny have been lost when comparing with *S. pombe* (fig. 2), presumably because of the highly reorganized chromosome structure to retain centromere function in fission yeasts (Rhind et al. 2011). Interestingly, we observed disomy of the second largest scaffold (385 kb) in *T. deformans* and not the corresponding syntenic scaffolds in other *Taphrina* species (fig. 3). Although it remains to be clear whether this is proper aneuploidy or duplications of a large part of a scaffolds,.aneuploidy is common in fungi and plays a role in increased virulence and better survival when cells undergo severe natural or induced environmental conditions (Kwon-Chung and Chang 2012). For the genes that have functional annotations, GO term enrichment revealed that the genes are mostly enriched in organic acid metabolic process (supplementary table S3, Supplementary Material online) and very interestingly also “leaf development” on this scaffold. Additionally, *Taphrina* spp. encode a single copy of a phosphoinositide-interacting protein (Td_0030400.1) and PFS2 (Td_0031400.1) on this scaffold of which the homologs in *Arabidopsis thaliana* are involved in leaf senescence and control of flowering time (Henderson et al. 2005; Xing et al. 2008), respectively. The exact mechanism by which aneuploidies confer an advantage to *T. deformans* is unclear, as we found no allelic differences in the additional scaffold copy and the median expression level of the scaffold is similar to the rest of scaffolds (supplementary fig. S3, Supplementary Material online). It remains to be determined whether aneuploidy is also apparent in isolates of different *Taphrina* species and whether the genes contained in these scaffolds have a role in interactions of the fungi with their hosts.

**Fig. 4.—:**
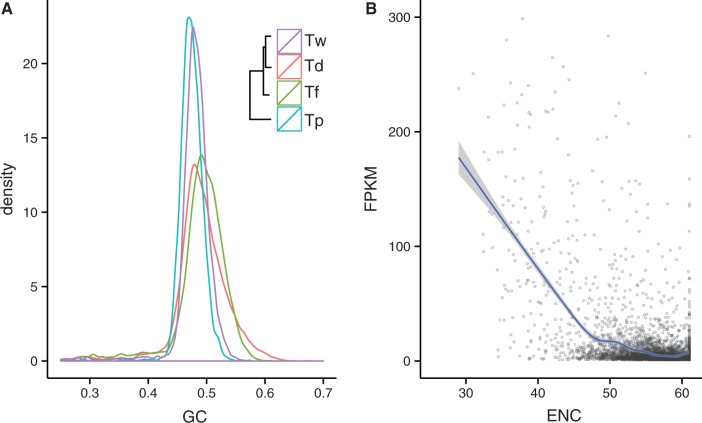
(*A*) Distribution of GC content in *Taphrina* species and (*B*) scatterplot showing relationship between codon usage (ENC) and expression count (FPKM) in *Taphrina wiesneri*.

The average GC contents of the *Taphrina* genomes range from 47.3% to 48.9%, which is higher than that of compared with other Taphrinomycotina members. Density plots of GC content in 2-kb windows of *Taphrina* genomes (fig. 4*A*) show a single peak suggesting that the GC content is evenly distributed along the scaffolds. Neither extreme GC nor AT-rich isochores were detected as observed in other yeasts. Interestingly, GC content profiles of *T. wiesneri* and *T. populina* are more similar and lower compared with other two species, which are in different positions in the phylogeny (fig. 4*A*). Consistent with our findings, *T. deformans* PYCC5710 also shows a higher average GC content. One of the major determinants of GC content in fungi is that more highly expressed genes preferentially use a set of codons that have particular GC biases (Bennetzen and Hall 1982). We first assessed codon usage by calculating the effective number of codons (ENC) and GC content at the third nucleotide positions (GC3) of codons across the four *Taphrina* proteomes. A gene that has a low ENC values indicates greater biases in codon usage, and we found the *Taphrina* genes show ENCs that were negatively correlated with higher expression values (fig. 4*B*, *P* value < 0.001). Additionally, we found that the GC3 was positively correlated with gene expression levels in all four *Taphrina* species (*P* value < 0.001). The interplay between codon usage and GC content is apparent in *Taphrina* species and is consistent with findings in other fungi (Hershberg and Petrov 2009). The two sets of GC profiles different to the *Taphrina* phylogenetic relationship may suggest similar gene expression profiles due to overlap in host environment during speciation of *Taphrina* genomes.

## *Taphrina* Proteome Specializations

**Fig. 5.—:**
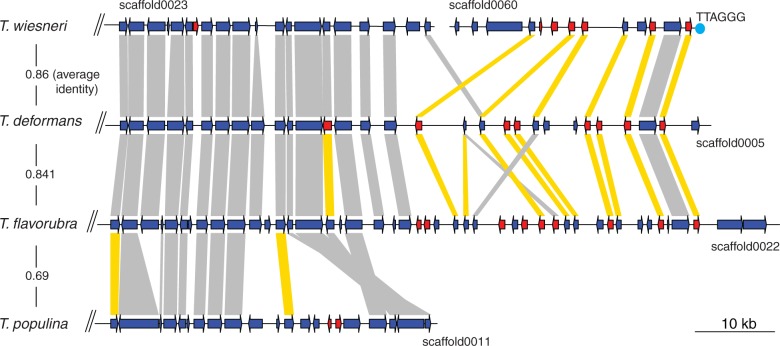
Divergent cluster in *Taphrina* species. Genes with signal peptide predicted are marked in red. Shaded bars connect homolog pairs as suggested by DAGchainer and are marked in yellow if divergence is less than 5% quantile of all possible pairwise comparisons. Average similarities between all homologs of pairwise species comparisons and putative telomeres are denoted.

We sought to identify specialized and expanded gene families in *Taphrina* species by first assigning Pfam domains to identify any genes with new combination of protein domains. Pairwise comparisons of domain numbers between the four *Taphrina* species show no significant differences, suggesting that the repertoire of annotated genes is similar across *Taphrina* species. Next, we sought to identify clusters of genes that are highly diverged within *Taphrina* spp. within syntenic regions, which have been shown to associate with virulence strength in other pathogenic fungi (Schirawski et al. 2010). Our analyses revealed a total of 58 regions containing 3–10 highly diverged genes adjacent to each other in all possible pairwise comparisons of four *Taphrina* species. Interestingly, the largest divergence cluster is enriched with genes encoding secreted products indicating their potential to be effectors, which are all located at scaffold ends with the *T. wiesneri* scaffold containing the telomeric repeat TTAGGG (fig. 5). This cluster is present in three out of four *Taphrina* species with no similar proteins found in *T. populina* assembly. Rapid expansion and diversification of gene families at subtelomeres in yeasts are not uncommon (Brown et al. 2010). It remains to be elucidated whether *Taphrina* utilize this region as the hotbeds for genomic innovation against plant immune response and the rapid diversification of secreted proteins as effectors that deals with specific hosts.

Predicted proteins from *Taphrina* genomes were clustered into a total of 10,356 gene families using OrthoMCL. Of those, 4,574 families contain at least one gene from *Taphrina* species and one from other fungi. A total of 1,324 families contain only genes from *Taphrina* members. For those that have functional annotation, topGO analysis suggests that the *Taphrina* species are particularly enriched in categories such as “regulation of transcription from RNA polymerase II promoter” (supplementary table S4, Supplementary Material online), which suggests that the mechanism for transcription regulation in *Taphrina* species may be diversified compared with other fungi. In each of the four *Taphrina* species, we also identified a single copy of perilipin, a protein responsible for lipid-based homeostasis in mammals, which was previously thought to be present only in pezizomycotinal ascomycetes (Bickel et al. 2009). Inhibition of perilipin like protein *MPL1* expression in the insect pathogen *Metarhizium anisopliae* shows reduced ability to breach insect cuticles (Wang and St Leger 2007). This suggests certain specialized genes that were involved in lipid metabolism have been found in *Taphrina* genomes and may be part of infection strategy. In the remainder of the Results section, we focus on topics that are of particular interest to *Taphrina* biology.

### 

#### Phosphorelay Signaling System through Taphrina Histidine Kinases

The two-component phosphorelay signaling system is one of the most extensively studied systems in fungi where they comprise histidine kinases (HKs), response regulators, and phosphotransfer proteins. Together these play important roles in osmosensing, oxidative stress, virulence, and even dimorphic switch between nonpathogenic to pathogenic states (Schaller et al. 2011). The model yeast *Sa. cerevisiae* and *S. pombe* encode one (SLN1) and three copies (MAK1/MAK2/MAK3) of HKs, respectively, and filamentous fungi in general possess an expanded repertoire of HKs allowing for different input into the phosphorelay. We identified 16–19 copies of HKs in the *Taphrina* genomes, a similar complement to the numbers found in filamentous fungi and more than any other fungal species in our selected proteomes (table 3; typically 1–19 copies in fungi as reviewed in Schaller et al. 2011). *Taphrina* species contain HKs with a range of domain combinations including both SLN1/MAK1 and MAK2/MAK3, suggesting that the two distinct osmo- and oxidative stress response pathways are present in *Taphrina* species, similar to fission yeast *S. pombe* (Quinn et al. 2011)*.* Additionally, all *Taphrina* species possess a single copy of an HK that contains an MHYT domain (Galperin et al. 2001), which is present in many bacterial HKs but not in our nine selected fungal proteomes. Searching through NCBI nr database reveals very few copies present in only the Pezizomycotina yeasts. Together they suggest the two component phosphorelay system in *Taphrina* are adapted to sense a diverse set of environment responses with a repertoire of HKs similar to many filamentous fungi some of which are also plant pathogens (Schaller et al. 2011).

**Table 3:** Summary of HKs Present in *Taphrina* and Seven Other Fungi Species Used in This Study

HK Family	*Fusarium graminearum*	*Sclerotinia sclerotiorum*	*Neurospora crassa*	*Saccharomyces cerevisiae*	*Schizosaccharomyces pombe*	*Ustilago maydis*	*Taphrina populina*	*T. flavorubra*	*T. deformans*	*T. wiesneri*
RIM15	1	1	1	1	1 ^a^	1	1	1	1	1
SSK1	1	2	2	1	1	4	1	1	1	1
SKN7	1	0	1	1	1	0	1	1	1	1
SLN1/MAK1 like	14	18	12	1	1	6	14	15	15	15
MAK2/MAK3 like	1	1	1	0	2	0	2	4	4	4
Total	15	19	13	1	3	6	16	19	19	19

Note.—*Pneumocystis jirovecii* contains 0 copies in all.

^a^Through literature search.

### Host Infection Signaling by Mating and Dimorphism-Related Genes

Changing from yeast to mycelium phases is critical for *Taphrina* fungi to establish an infection in the host (Mix 1935). The triggers and molecular mechanisms of dimorphism in *Taphrina* remain unclear, but ploidy change by mating and/or host signals is likely to be important as described in *U. maydis* (Nadal et al. 2008). We identified homologs of most of the *S. pombe* sex-related genes in the four *Taphrina* species (supplementary table S5, Supplementary Material online). Those include genes involved in mating type switching (for instance, *swi1*, *swi5*, *swi6*, and *swi10*) and genes in the pheromone-activated MAPK signaling pathway (e.g., *bry1* and *bry2*). Interestingly, Rik1-associated factor 1 (*raf-1*) and meiosis protein *mei2* are absent only in *T. populina.* Mei2 is a RNA-binding protein that is important for induction of meiosis in *S. pombe* (Sherwood and Bennett 2009), suggesting that a Mei2-indipendent pathway may exist for activation of meiosis in *T*. *populina*.

### Invasion through Cell Wall Degradation

The plant cell wall represents the primary barrier to most plant pathogens and CAZymes (carbohydrate active enzymes) that are able to break down plant cell wall are thus important for plant pathogens in establishing infection or in accessing nutrients. CAZymes are categorized into different classes and families in the CAZy database (http://www.cazy.org, last accessed April 5, 2014) including glycoside hydrolases (GH), glycosyl transferases, polysaccharide lyases, carbohydrate esterases, and carbohydrate-binding modules (Cantarel et al. 2009). The high diversity of CAZymes in *Taphrina* species suggest that they target a wide aspect of plant cell wall components including cellulose, hemicellulose, and pectin (supplementary table S6, Supplementary Material online). The genomes of the four *Taphrina* species contain 226–237 copies of putative of CAZymes, which is greater than other members in Taphrinomycotina but smaller than other plant parasitic fungi including the necrotrophic *Sc. sclerotiorum* and hemibiotrophic *F. graminearum* (supplementary table S6, Supplementary Material online). This observation is consistent with a previous study that suggests biotrophic fungi tend to have fewer CAZymes than necrotrophic or hemibiotrophic fungi (Zhao et al. 2013). In addition, *Taphrina* species also have GH enzymes that are active to fungal cell wall components including β-1,3 glucan and chitin. The four *Taphrina* species share similar repertories of CAZymes to each other but some species specific differences are apparent especially in *T. populina.* For instance, most of the GH families such as GH38, GH53, and GH79 are shared among all four species, but *T. populina* lacks some GH families that are present in all other three species (supplementary table S7, Supplementary Material online), which may relate to differences in host specificity. For instance, GH30, GH32, and GH10 genes are not found in *T. populina* but present in other two or three *Taphrina* species with signal peptides and may secret into host tissues to establish infections. In particular, GH10 proteins have ability to degrade xylan, a main component of secondary plant cell wall, which may relate to *T. populina*’s relatively weak symptom and localization on leaves.

### Imbalance of Plant Hormones in Infected Tissues Caused by Pathogens

The distinctive characteristics of the plant symptoms caused by *Taphrina* species are deformation of plant tissues. These deformations are probably due to induced imbalances of plant hormones in the host tissues. In particular, production of auxin and IAA by fungi is thought to be causally related to the symptoms. An IAA biosynthesis pathway via indole-3-acetaldehyde (IAAld pathway: Tryptophan–Indole-3-pyrvic acid–IAAld–IAA) has been identified in the smut fungus *U. maydis* as the IAA main biosynthesis pathway. Each of the four *Taphrina* species contains orthologs of genes involved in this fungal IAA biosynthetic pathway; two orthologs of the tryptophan aminotranferases (encoded by *Tam* genes) and two orthologs of indole-3-acetaldehyde dehydrogenates (encoded by *iad* genes) (supplementary table S8, Supplementary Material online). This suggests that *Taphrina* spp. have a similar fungal IAA biosynthesis pathway to *U. maydis* (Reineke et al. 2008). Interestingly, putative orthologs of a plant YUC gene (Mashiguchi et al. 2011), which encodes flavin monooxygenase and is involved in plant IAA biosynthesis, were found in all four *Taphrina* species (one orthologs in *T. populina* and two orthologs in the three other species) (supplementary table S8, Supplementary Material online). Although *Arabidopsis* tryptophan aminotransferase gene (TAA1) ortholog is not present, tryptophan aminotransferase genes (*Tam*) are present in *Taphrina* species as stated above, suggesting the *Taphrina* species may have another IAA biosynthesis pathway (Tryptophan–Indole-3-pyrvic acid–IAA) resembling that used by the plants in addition to the fungal IAAld pathway (Mashiguchi et al. 2011)*.* No orthologs of tryptophan monooxygenase genes or indole acetamide hydrolase genes were found in *Taphrina* species, suggesting the fungi did not have the indole-acetamide pathway that is present in bacteria (e.g., *Agrobacterium*). In addition, no orthologs of IAA conjugate-related genes (e.g., *ILr1*, *IAR3*, *ILL1*, *ILL2*, *GH3*, and *UGT84B1*) were present in *Taphrina* species, suggesting that auxin conjugates were not associated with IAA imbalance in host plants (Woodward and Bartel 2005).

Cytokinin also may be responsible for the deformation symptoms, and cytokinins production by *Taphrina* species has been reported (Johnston and Trione 1974; Sziraki et al. 1975). Each *Taphrina* species harbored putative orthologs of principal cytokinin biosynthesis genes, that is, tRNA-isopentenyltransferase (encoded by *tRNA-IPT*), cytokinin hydroxylases (encoded by *CYP735A* gene), and phosphoribohydrolases (encoded by *LOG* gene) (supplementary table S8, Supplementary Material online), confirming the ability of *Taphrina* species to produce cytokinin. We also identified putative orthologs of GA conversion-related genes (*GA2ox*, *GA3oc*, and *GA20ox*) and G4-desaturase (Hedden et al. 2001) in three of the four species (supplementary table S8, Supplementary Material online), suggesting that these genes may have a role in converting GAs in plant tissues during the colonization by *Taphrina* species. However, interestingly, no orthologs of those gibberellin-related genes were identified in *T. populina,* which may be related to the less severe symptom by the fungus than other three species.

Putative genes involved in abscisic acid (ABA) biosynthesis have been identified in *T. deformans* (Cissé et al. 2013), although the ABA production capability of the *Taphrina* species remains to be confirmed. In this study, we also found that each *Taphrina* species contain orthologs of the *Botrytis cinerea* ABA biosynthesis genes, for example, P450 monooxygenase gene (*BcABA1*, *BcABA2*) and short chain dehydrogenase/reductase gene (*BcABA4*) (Siewers et al. 2006) (supplementary table S8, Supplementary Material online). However, *BcABA3* was not found in *Taphrina* species. The role of BcABA3 in ABA biosynthesis pathway is still unknown. In addition, each *Taphrina* species genome harbored orthologous copies of plant ABA biosynthesis genes, for example, xanthoxin dehydrogenase gene, 9-*cis*-epoxycarotenoid dioxygenase gene, and abscisic-aldehyde oxidase gene but lacked a neoxanthin synthase gene (supplementary table S8, Supplementary Material online). Together they suggest all *Taphrina* species studied can synthesize ABA but are unable to synthesize neoxanthin.

## Conclusion

In addition to their status as important plant pathogens, the *Taphrina* yeasts are also of particular evolutionary interest as they form part of an ancient clade within the Ascomycete fungi. Unlike its related fission yeast genus *Schizosaccharomyces,* the *Taphrina* fungi display a high degree of gene colinearity. Our comparative analysis of the *Taphrina* genome structures has revealed biology that shows these organisms use various strategies similar to those used by yeasts to adapt to the host environment. For example, aneuploidy results in additional copies of genes that may have host-modulating roles, whereas secreted proteins that may be putative effectors are highly diverged between *Taphrina* species are located in subtelomeres. We have also highlighted a few potential areas that could follow. First, the most closely related *Taphrina* perilipin and MHYT HK orthologs in ascomycetes were found only in the Pezizomycotina subphylum but neither Taphrinomycotina nor Saccharomycotina, suggesting that the origin of these genes in *Taphrina* may be obtained via horizontal gene transfer. More genome sequences in the order of Taphrinales are needed to confirm this observation. Second, although inoculation and recovering of some *Taphrina* fungi in hosts still remain technically difficult, infection assays with divergent gene clusters deletion lines would demonstrate whether these genes attribute to virulence in hosts. Finally, the most interesting topic may be the copy number differences in repertoires of genes that may modulate plant hormone production. It remains to be elucidated how these genes are regulated between *Taphrina* species to cause differences in degrees of plant deformity. As these results demonstrate, comparative analyses make these *Taphrina* genome sequences a valuable resource for application of further omics technologies and the evolutionary studies on early evolution of yeasts.

## Supplementary Material

Supplementary figures S1–S3 and tables S1–S9 are available at *Genome Biology and Evolution* online (http://www.gbe.oxfordjournals.org/).

Supplementary Data
