# Supplementary material for: Comparative Genomics of Taphrina Fungi Causing Varying Degrees of Tumorous Deformity in Plants
Source: Genome Biol Evol. 2014 Mar 28;6(4):861–72. doi: 10.1093/gbe/evu067 (PMC4007546; doi:10.1093/gbe/evu067)
Supplement: Supplementary Data [file supp_6_4_861__index.html]

Comparative genomics of Taphrina fungi causing varying degrees of tumourous deformity in plants — Comparative Genomics of Taphrina Fungi Causing Varying Degrees of Tumorous Deformity in Plants — Supplementary Data 

# Comparative Genomics of *Taphrina* Fungi Causing Varying Degrees of Tumorous Deformity in Plants

## Supplementary Data

files

**Files in this Data Supplement:**

- Supplementary Data - pdf file
- Supplementary Data - pdf file
