# Supplementary material for: Comparative Genomics of Taphrina Fungi Causing Varying Degrees of Tumorous Deformity in Plants
Source: Genome Biol Evol. 2014 Mar 28;6(4):861–72. doi: 10.1093/gbe/evu067 (PMC4007546; doi:10.1093/gbe/evu067)

Figure S1. A) Mitochondrial genome of *T. wiesneri* and B) mitochondrial gene orders between the four *Taphrina* species

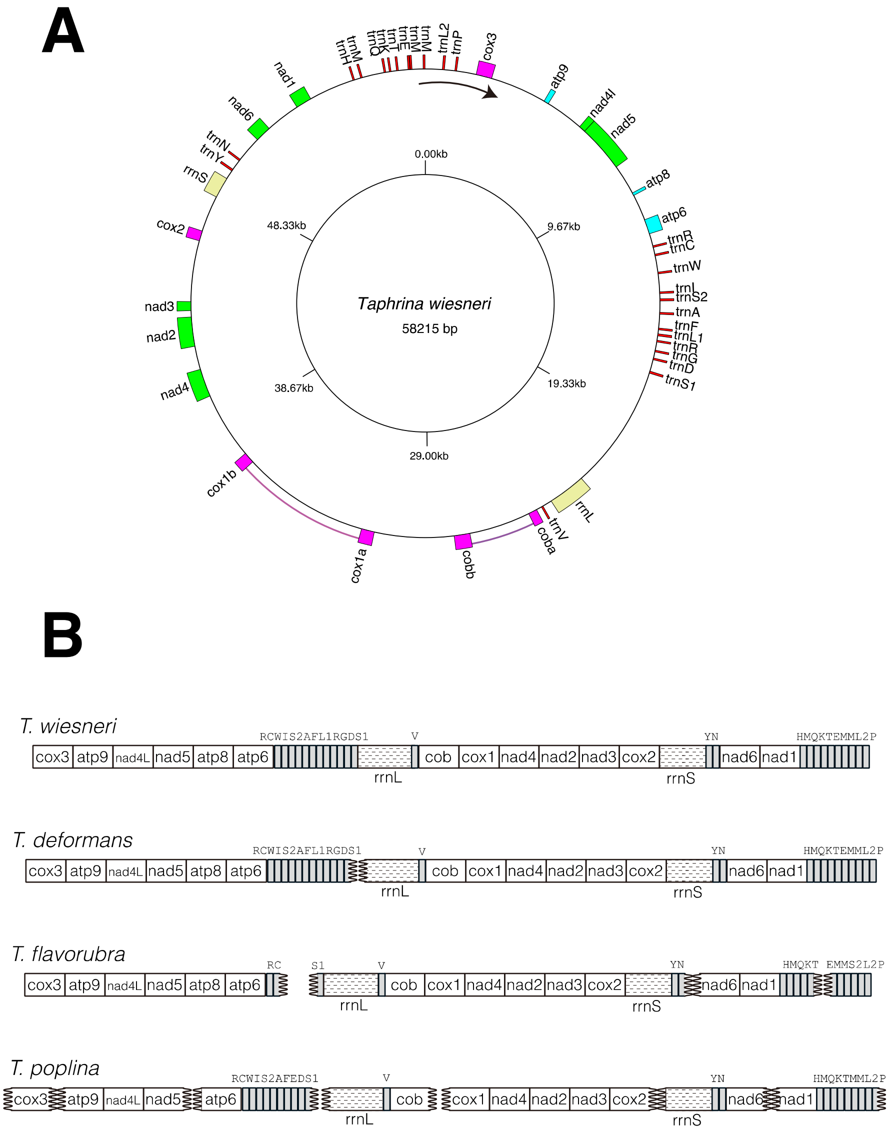

Figure S2. Dotplots showing the relationship between the four *Taphrina* species using *T. wiesneri* as the main comparator. For reference, a subset of the global fungi phylogeny is also plotted.

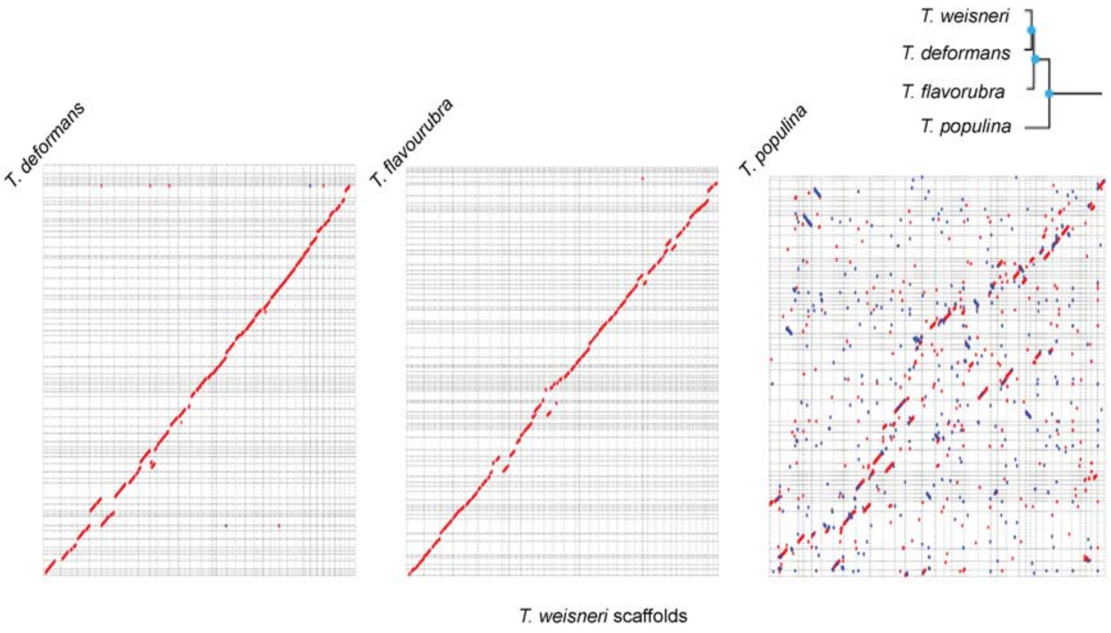

Figure S3. Boxplot showing the expression levels of genes in the largest ten scaffolds of *T. deformans*. The disomy scaffold is marked in yellow.

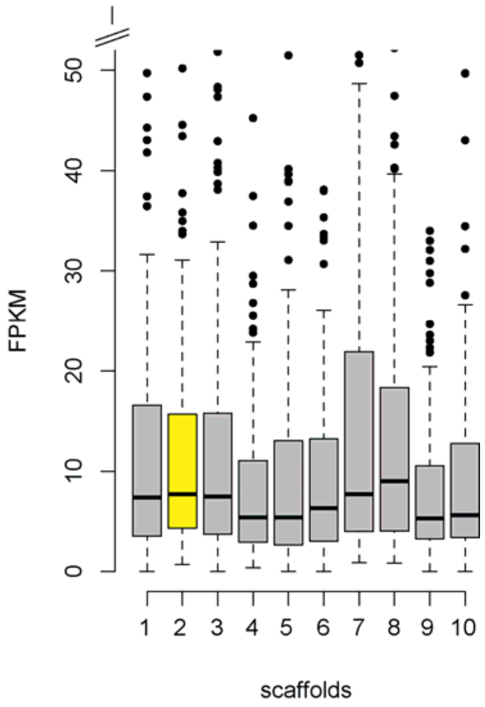

Supplement: Supplementary Data [file supp_evu067_Taphrina.SupplementaryFigures.v1.2.pdf]
