# Supplementary material for: Comparative Genomics of Taphrina Fungi Causing Varying Degrees of Tumorous Deformity in Plants
Source: Genome Biol Evol. 2014 Mar 28;6(4):861–72. doi: 10.1093/gbe/evu067 (PMC4007546; doi:10.1093/gbe/evu067)
Supplement: Supplementary Data [file supp_evu067_Taphrina.SupTables.revisedv2.pdf]

Table S1 – *Taphrina* species used in this study

Species	JCM no.	Source of isolation	Host range	Distribution	Disease symptom
T. wiesneri	22204	Sakura cherry (isolated by K. Tubaki)	*Prunus* (Subgenus Cerasus)	Europe, North America, Australia, New Zealand, South Africa, Japan	Witchs' brooms
T. deformans	22205	Leaf of *Prunus persica* in Minami-Senri, Oasaka, Japan (isolated by T. Yokoyama in 1968 March)	*Prunus* (Subgenus Amygdalus)	Worldwide	Leaf curl
T. populina	22190	*Populus nigra* (isolated by A.J. Mix)	*Populus*	Europe, North America, India, China, Japan	Yellow leaf spots
T. flavorubra	22207*	Unknown (isolated by C.L. Kramer)	*Prunus* (Subgenus Prunus)	North America	Deformed fruits (pockets)

* The strain was identified by C.L. Kramer as *T. flavorubra* , although it has 2 nucleotide substitutions in D1/D2 region of LSU rDNA compared to *T. flavorubra* NRRL Y-17795^{T} (AF492040).

Table S2 – *Taphrina* genome repeat content

Species	Category	copy number	total length of repeats in genome	number of copies	% assembly
T. wiesneri	DNA	17	24,376	81 (54)	0.2%
	LINE	1	552	1 (1)	0.0%
	LTR	14	21,303	30 (20)	0.2%
	no TE feature	1	9,089	34 (30)	0.1%
	**Total**	**33**	**55,320**	**146 (105)**	**0.4%**
T. deformans	DNA	33	82,688	209 (97)	0.6%
	LINE	4	4,324	5 (4)	0.0%
	LTR	42	186,615	245 (107)	1.4%
	no TE feature	9	352,907	863 (168)	2.6%
	**Total**	**88**	**626,534**	**1,322 (376)**	**4.5%**
T. populina	DNA	19	55,608	211 (51)	0.5%
	LINE	10	14,726	36 (26)	0.1%
	LTR	60	112,280	402 (87)	0.9%
	no TE feature	8	79,800	320 (139)	0.7%
	**Total**	**97**	**262,414**	**969 (303)**	**2.2%**
T. flavorubra	DNA	52	122,064	336 (142)	0.8%
	LINE	11	51,413	85 (37)	0.3%
	LTR	88	466,814	760 (299)	3.0%
	no TE feature	22	538,156	1,540 (460)	3.4%
	**Total**	**173**	**1,178,447**	**2,721 (938)**	**7.5%**

Table S3 - List of significantly enriched GO terms on the disomy scaffold of *T. deformans* (p< 0.05)

GO terms	GO term definition	Annotated	Significant	Expected	p values
GO:0006082	organic acid metabolic process	370	18	12.97	0.0012
GO:0045899	positive regulation of RNA polymerase II transcriptional preinitiation complex assembly	3	2	0.11	0.0036
GO:0051004	regulation of lipoprotein lipase activity	3	2	0.11	0.0036
GO:0043666	regulation of phosphoprotein phosphatase activity	4	2	0.14	0.007
GO:0008054	cyclin catabolic process	4	2	0.14	0.007
GO:0048366	leaf development	5	2	0.18	0.0114
GO:0045842	positive regulation of mitotic metaphase/anaphase transition	6	2	0.21	0.0167
GO:0006303	double-strand break repair via nonhomologous end joining	6	2	0.21	0.0167
GO:0034765	regulation of ion transmembrane transport	6	2	0.21	0.0167
GO:0006783	heme biosynthetic process	12	3	0.42	0.0227
GO:0044282	small molecule catabolic process	51	3	1.79	0.0299
GO:0007091	mitotic metaphase/anaphase transition	37	5	1.3	0.0345
GO:0045937	positive regulation of phosphate metabolic process	18	3	0.63	0.0348
GO:0043331	response to dsRNA	10	2	0.35	0.0349
GO:0030162	regulation of proteolysis	12	2	0.42	0.0349
GO:0045839	negative regulation of mitosis	20	2	0.7	0.035
GO:0034641	cellular nitrogen compound metabolic process	1806	63	63.32	0.0355
GO:0007067	mitosis	112	9	3.93	0.0409
GO:0016567	protein ubiquitination	91	9	3.19	0.0456

Table S4. GO terms enriched in novel or diverged families identified in each *Taphrina* species

Species	GO terms	GO term definition	Annotated	Significant	Expected	p values
T. wiesneri	GO:0006357	regulation of transcription from RNA polymerase II promoter	544	59	34.28	0.00051
T. wiesneri	GO:0009688	abscisic acid biosynthetic process	5	3	0.32	0.00226
T. wiesneri	GO:0009749	response to glucose stimulus	6	3	0.38	0.00431
T. wiesneri	GO:0000272	polysaccharide catabolic process	86	9	5.42	0.00701
T. deformans	GO:0006357	regulation of transcription from RNA polymerase II promoter	225	69	36.59	1.10E-08
T. deformans	GO:0006355	regulation of transcription, DNA-dependent	547	152	88.96	1.40E-08
T. deformans	GO:0001539	ciliary or flagellar motility	4	4	0.65	0.0007
T. deformans	GO:0007010	cytoskeleton organization	111	21	18.05	0.00411
T. deformans	GO:0007155	cell adhesion	22	8	3.58	0.00426
T. flavorubra	GO:0006355	regulation of transcription, DNA-dependent	593	186	123.36	7.60E-09
T. flavorubra	GO:0006357	regulation of transcription from RNA polymerase II promoter	238	74	49.51	5.60E-06
T. flavorubra	GO:0009164	nucleoside catabolic process	99	17	20.6	0.00048
T. flavorubra	GO:0007599	hemostasis	4	4	0.83	0.00187
T. flavorubra	GO:0080111	DNA demethylation	4	4	0.83	0.00187
T. flavorubra	GO:0000270	peptidoglycan metabolic process	4	4	0.83	0.00187
T. flavorubra	GO:0050817	coagulation	4	4	0.83	0.00187
T. flavorubra	GO:0042060	wound healing	4	4	0.83	0.00187
T. flavorubra	GO:0006486	protein glycosylation	63	21	13.11	0.00193
T. flavorubra	GO:0009190	cyclic nucleotide biosynthetic process	7	5	1.46	0.00557
T. flavorubra	GO:0006334	nucleosome assembly	45	17	9.36	0.00746
T. flavorubra	GO:0042742	defense response to bacterium	10	6	2.08	0.00775
T. flavorubra	GO:0009401	phosphoenolpyruvate-dependent sugar phosphotransferase system	5	4	1.04	0.00778
T. flavorubra	GO:0001822	kidney development	5	4	1.04	0.00778
T. flavorubra	GO:0035295	tube development	7	6	1.46	0.00893
T. flavorubra	GO:0048481	ovule development	3	3	0.62	0.00898
T. flavorubra	GO:0030244	cellulose biosynthetic process	3	3	0.62	0.00898
T. flavorubra	GO:0060003	copper ion export	3	3	0.62	0.00898
T. flavorubra	GO:0042181	ketone biosynthetic process	3	3	0.62	0.00898
T. flavorubra	GO:0042542	response to hydrogen peroxide	8	4	1.66	0.00899
T. populina	GO:0034644	cellular response to UV	11	4	0.71	0.0039

Table S5. Sex related genes in *Taphrina* species

Gene name	Gene description	*T. wiesneri*	*T. deformans*	*T. flavorubra*	*T. poplina*
ago1	Protein argonaute	Tw_0427400.1	Td_0235200.1	Tf_0142500.1	Tp_0501700.1
byr1	MAPK kinase	Tw_0131200.1	Td_0354000.1	Tf_0338900.1	Tp_0131000.1
byr2-like	MAPK kinase kinase	Tw_0001800.1	Td_0501100.1	Tf_0322900.1	Tp_0170600.1
clr4	Histone-lysine N-methyltransferase, H3 lysine-9 specific	Tw_0541300.1	Td_0044000.1	Tf_0073900.1	Tp_0453400.1
dcr1-like	Protein Dicer	Tw_0256600.1	Td.scaffold0022 (4387-5675)	Tf_0249600.1	Tp_0058700.1
mam1	Mating factor M secretion protein mam1	Tw_0336500.1	Td_0378200.1	Tf_0571600.1	Tp_0018100.1
matmc_1	Mating-type M-specific polypeptide Mc	Tw_0472400.1	Td_0585100.1	Tf.scaffold0055 (34384-36194)	Tp_0379800.1
matPi	Mating-type P-specific polypeptide Pi	Tw_0274000.1	Td_0029700.1	Tf_0012300.1	Tp_0255900.1
mei2-like	Meiosis protein mei2	Tw_0218600.1	Td_0414900.1	Tf.scaffold0093 (22798-23411)	ND
msh3-like	Mating-type switching protein swi4	Tw_0035800.1	Td_0372400.1	Tf_0063800.1	Tp_0073700.1
spk1	Mitogen-activated protein kinase spk1	Tw_0113900.1	Td_0016300.1	Tf_0216300.1	Tp_0348400.1
rad22	DNA repair protein RADC (RAD52 family)	Tw_0597700.1	Td_0115900.1	Tf_0460600.1	Tp_0117500.1
rhp51	DNA repair protein rhp51 (RAD51 homolog)	Tw_0518700.1	Td_0279500.1	Tf_0242600.1	Tp_0065400.1
raf1	Rik1-associated factor 1	Tw_0487000.1	Td_0271600.1	Tf_0111100.1	ND
ras1	Ras-like protein 1	Tw_0357100.1	Td_0310000.1	Tf_0378400.1	Tp_0162600.1
rhp6	Ubiquitin-conjugating enzyme E2 2	Tw_0398700.1	Td_0158800.1	Tf_0163800.1	Tp_0186900.1
slm9	Histone transcription regulator slm9	Tw_0293500.1	Td_0539200.1	Tf_0441200.1	Tp_0273300.1
sir2	NAD-dependent histone deacetylase sir2	Tw_0518900.1	Td_0279300.1	Tf_0242800.1	Tp_0065200.1
map3	Pheromone M-factor receptor	ND	ND	ND	ND
ste11	Transcription factor ste11	Tw_0328200.1	Td_0356600.1	Tf_0098200.1	Tp_0204900.1
shk1	Serine/threonine-protein kinase shk1/pak1	Tw_0179900.1	Td_0407600.1	Tf_0231600.1	Tp_0098300.1
swi1	Mating-type switching protein swi1	Tw_0016200.1	Td_0229000.1	Tf_0487500.1	Tp_0149300.1
sap1	switch-activating protein Sap1	ND	ND	ND	ND
swi2	Swi5 complex subunit Swi2	ND	ND	ND	ND
swi5	Mating-type switching protein swi5	Tw_0010600.1	Td_0224100.1	Tf_0332500.1	Tp_0180000.1
swi6	Chromatin-associated protein swi6	ND	ND	ND	ND
swi10	Mating-type switching protein swi10	Tw_0026000.1	Td_0611500.1	Tf_0274300.1	Tp_0239500.1
msh3	MutS protein homolog 3	Tw_0035800.1	Td_0372400.1	Tf_0063800.1	Tp_0073700.1
clr1	cryptic loci regulator Clr1	ND	ND	ND	ND

ND; not detected

Table S6. Comparisons of the numbers of CAZymes in *Taphrina* species and other fungi

	Tw	Td	Tf	Tp	Sp	Pj	Sc	Fg	Nc	Ss	Um
CBM	15	14	14	14	6	4	10	69	45	66	10
CE	43	48	45	48	17	7	18	140	65	99	62
GH	88	87	85	84	56	11	58	253	182	223	108
GT	90	81	82	80	61	34	68	109	83	92	67
PL	1	1	1	0	0	0	0	17	4	5	3
Total	237	231	227	226	140	56	154	588	379	485	250

Td: *T. deformans* , Tw: *T. wiesneri* , Tf: *T. flavorubra* , Tp: *T. populina* ,
Sp: S. pombe, Pj: Pneumocystis jirovecii, Sc: S. cerevisiae, Fg: Fusarium graminearum, Nc: Neurospora crassa, Ss: Sclerotinia sclerotiorum, Um: Ustilago maydis.
CBM: Carbohydrate-Binding Modules, CE: Carbohydrate Esterases, GH: Glycoside Hydrolases, GT: GlycosylTransferases, PL: Polysaccharide Lyases

Table S7. Gene numbers of each Cazyme family in Fungi

Cazyme family	Tw	Td	Tf	Tp	Sp	Pj	Sc	Fg	Nc	Ss	Um	Known Substrate
CBM1	0	0	0	0	1	0	0	11	17	19	0	
CBM13	0	0	0	0	0	0	0	4	3	3	2	
CBM16	0	0	0	0	0	0	0	4	0	0	0	
CBM17	0	0	0	0	0	0	0	0	0	1	0	
CBM18	0	0	0	0	0	0	1	11	4	12	1	
CBM19	1	1	1	1	0	0	1	0	0	0	0	
CBM20	1	1	1	1	0	0	0	1	3	5	0	
CBM21	1	1	1	1	0	0	4	2	1	1	0	
CBM22	0	0	0	0	0	0	0	3	0	0	0	
CBM23	0	0	0	0	1	0	0	0	0	0	0	
CBM24	0	0	0	0	0	0	0	2	4	6	0	
CBM27	0	1	1	1	1	0	0	0	0	0	0	
CBM32	1	1	0	1	0	0	1	2	1	0	0	
CBM35	0	0	0	0	0	0	0	2	1	1	1	
CBM37	1	0	0	0	0	0	0	0	0	1	0	
CBM38	0	0	0	0	0	0	0	0	1	0	0	
CBM39	0	0	0	0	0	1	0	0	0	0	0	
CBM4	0	0	0	0	0	0	0	0	0	0	1	
CBM42	0	0	0	0	0	0	0	1	1	1	0	
CBM43	1	1	1	1	2	1	2	1	1	1	1	
CBM46	0	0	0	0	0	0	0	0	0	1	0	
CBM48	1	1	1	1	0	1	1	1	1	2	1	
CBM50	4	3	4	3	0	0	0	9	5	6	2	
CBM51	0	0	0	0	0	0	0	0	0	1	0	
CBM52	4	4	4	3	1	1	0	0	1	0	0	
CBM53	0	0	0	1	0	0	0	0	0	0	0	
CBM6	0	0	0	0	0	0	0	1	0	1	0	
CBM61	0	0	0	0	0	0	0	5	0	2	0	
CBM63	0	0	0	0	0	0	0	3	0	1	1	
CBM66	0	0	0	0	0	0	0	1	0	0	0	
CBM67	0	0	0	0	0	0	0	4	1	1	0	
CBM9	0	0	0	0	0	0	0	1	0	0	0	
CE1	11	15	13	11	6	3	10	29	18	17	14	
CE10	21	20	21	25	8	3	3	66	26	42	31	
CE11	0	0	0	0	0	0	1	0	0	0	0	
CE12	2	3	2	2	0	0	1	5	2	5	0	
CE13	0	0	0	0	0	0	0	0	0	0	2	
CE14	1	0	1	2	1	0	1	0	1	1	1	
CE15	0	0	0	0	0	0	0	0	1	2	0	
CE16	2	2	2	1	0	0	0	5	2	6	0	
CE2	0	1	0	0	0	0	0	1	1	1	0	
CE3	2	1	1	1	1	0	0	7	5	4	0	
CE4	1	1	1	1	1	0	2	9	4	5	8	
CE5	2	3	3	4	0	0	0	13	3	8	4	
CE6	0	0	0	0	0	0	0	0	0	0	0	
CE7	0	1	0	0	0	1	0	1	0	1	0	
CE8	1	1	1	1	0	0	0	3	1	5	1	PCW (Pectin)
CE9	0	0	0	0	0	0	0	1	1	2	1	
GH1	0	0	0	1	0	0	0	3	1	3	0	CW (b-glucans)
GH10	1	1	0	0	0	0	0	5	4	2	2	PCW (hemicellulose)
GH105	1	1	1	1	0	0	0	3	2	1	2	PCW (Pectin)
GH106	1	1	1	1	0	0	0	1	0	1	0	
GH108	0	0	0	0	0	0	0	0	1	0	0	
GH109	3	3	3	3	3	0	1	15	4	6	6	
GH11	0	0	0	0	0	0	0	2	2	3	1	PCW (hemicellulose)
GH114	1	1	1	2	0	0	0	2	1	1	0	
GH115	0	0	0	0	0	0	0	2	1	1	1	PCW (hemicellulose)
GH12	1	1	1	1	0	0	0	4	1	4	0	PCW (celllulose)
GH121	0	0	0	0	0	0	0	1	1	0	0	
GH125	2	2	2	2	1	0	0	3	3	3	1	PG (N-glycans)
GH127	0	0	0	0	0	0	0	2	0	0	0	
GH128	6	5	5	5	1	0	0	4	3	3	5	
GH13	7	8	8	8	12	1	8	4	9	9	3	FCW + ESR (a-glucans)
GH131	0	0	0	0	0	0	0	1	3	2	0	
GH132	2	2	2	2	3	1	5	2	2	0	0	
GH15	1	1	1	1	2	0	1	2	2	4	1	ESR (a-glycans)
GH16	16	14	14	13	3	0	4	24	15	18	20	FCW (b-glycans)
GH17	2	2	3	3	1	0	4	3	4	6	2	FCW (b-1,3-glucan)
GH18	3	3	3	3	1	0	2	18	12	14	4	FCW (chitin)
GH2	0	0	0	0	0	0	0	10	5	2	1	CW (b-glucans)
GH20	1	1	1	1	0	0	0	3	1	1	2	FCW (chitin)
GH23	0	0	0	0	0	0	0	1	0	2	2	BPG
GH25	0	0	0	0	0	0	0	0	1	1	1	BPG
GH26	0	0	0	0	0	0	0	0	1	1	1	BPG
GH27	0	0	0	0	1	0	0	2	0	3	1	PCW (hemicellulose)
GH28	3	3	3	1	0	0	1	6	2	16	1	PCW (Pectin)
GH29	0	0	0	0	0	0	0	1	0	0	0	PCW (hemicellulose)
GH3	3	3	2	2	1	0	0	21	10	13	3	CW (b-glucans)
GH30	1	1	1	0	0	0	0	0	2	0	2	FCW
GH31	2	3	3	2	4	1	1	8	5	6	3	PG + ESR + PCW (hemicellulose)
GH32	1	1	1	0	2	1	1	6	1	1	2	ESR (sucrose/inulin)
GH33	0	0	0	0	0	1	2	1	0	1	0	
GH35	0	0	0	0	0	0	0	3	2	4	1	PCW (hemicellulose)
GH36	0	0	0	0	0	0	0	2	0	0	0	PCW (hemicellulose)
GH37	2	2	2	2	1	1	2	2	2	1	2	ESR (trehalose)
GH38	0	0	0	1	1	0	1	1	1	1	2	PG (N-/O-glycans)
GH4	0	0	0	0	0	0	0	1	1	0	0	ESR
GH42	0	0	0	0	0	0	0	0	0	0	1	
GH43	0	0	0	0	0	0	0	19	7	5	2	PCW (pectin + hemicellulose)
GH44	0	0	0	0	0	0	0	0	0	0	0	
GH45	0	0	0	0	0	0	0	1	1	2	3	PCW (celllulose)
GH47	4	4	4	4	2	1	3	6	8	8	3	PG (N-/O-glycans)
GH5	8	9	7	7	3	0	4	14	6	14	12	PCW (cellulose + hemicellulase)
GH51	0	0	0	0	0	0	0	2	1	2	2	PCW (hemicellulose)

GH53	0	0	0	1	0	0	0	1	1	2	0	PCW (hemicellulose)
GH54	0	0	0	0	0	0	0	1	1	1	0	PCW (hemicellulose)
GH55	1	1	1	1	0	0	0	3	6	4	1	FCW (b-1,3-glucan)
GH6	0	0	0	0	0	0	0	1	3	1	0	PCW (celllulose)
GH62	0	0	0	0	0	0	0	1	0	0	1	PCW (hemicellulose)
GH63	2	2	2	2	1	1	2	1	1	1	1	PG (N-glycans)
GH64	0	0	0	0	0	0	0	2	2	2	0	CW (β-1,3-glucan)
GH65	0	0	0	0	0	0	1	0	0	1	0	ESR (trehalose)
GH67	0	0	0	0	0	0	0	1	1	0	0	PCW (hemicellulose)
GH7	0	0	0	0	0	0	0	2	5	3	0	PCW (celllulose)
GH71	2	2	2	2	2	0	0	0	5	8	0	FCW (b-1,3-glucan)
GH72	4	4	4	4	4	1	5	3	5	6	1	FCW (b-1,3-glucan)
GH73	0	0	0	0	0	0	0	1	0	0	0	
GH74	1	1	1	1	1	0	6	2	2	1	1	PCW (celllulose)
GH75	0	0	0	0	0	0	0	0	1	1	0	FCW (chitin)
GH76	1	2	1	1	4	1	2	8	10	12	1	FCW (chitin)
GH78	1	0	2	0	0	0	0	7	1	4	0	PCW (Pectin)
GH79	0	0	0	3	0	0	0	1	2	2	1	
GH8	0	0	0	0	0	0	0	0	0	0	1	CW
GH81	2	2	2	2	2	1	2	1	1	1	0	FCW (b-1,3-glucan)
GH85	0	0	0	0	0	0	0	0	0	0	1	FCW
GH88	0	0	0	0	0	0	0	1	0	0	0	PCW (pectin)
GH89	0	0	0	0	0	0	0	0	0	1	0	FCW
GH9	0	0	0	0	0	0	0	0	1	0	1	CW
GH92	1	1	1	1	0	0	0	0	2	5	3	
GH93	0	0	0	0	0	0	0	2	3	1	0	PCW (hemicellulose)
GH94	0	0	0	0	0	0	0	0	1	0	0	PCW (celllulose)
GH95	0	0	0	0	0	0	0	2	0	2	0	
GH99	1	0	0	0	0	0	0	0	0	0	0	
GT1	1	1	1	1	0	0	1	14	4	7	3	
GT15	4	4	4	4	6	0	9	3	5	4	2	
GT17	1	0	1	0	0	0	0	1	1	2	1	
GT2	14	14	12	13	3	2	4	18	12	18	16	
GT20	3	3	3	3	5	2	4	3	3	3	3	
GT21	3	2	2	2	0	1	0	1	1	1	1	
GT22	4	4	4	4	4	2	4	4	3	3	4	
GT23	2	0	0	0	0	0	0	0	0	0	0	
GT24	1	1	1	1	1	1	1	1	1	1	1	
GT25	1	1	2	1	0	0	0	1	2	3	0	
GT26	0	0	1	0	0	0	0	1	0	0	0	
GT28	2	2	2	2	1	1	1	1	0	1	1	
GT29	0	0	0	1	0	0	0	0	0	0	0	
GT3	1	1	1	1	0	1	2	1	1	1	1	
GT31	3	0	2	1	1	0	0	2	2	2	3	
GT32	9	10	9	9	5	2	4	6	5	5	2	
GT33	1	1	1	1	1	2	1	1	1	1	1	
GT34	4	3	3	2	7	0	2	5	3	2	0	
GT35	0	0	0	0	0	1	1	1	1	1	0	
GT39	3	3	3	3	3	4	7	3	4	3	3	
GT4	4	4	4	4	3	4	3	5	6	5	4	
GT41	0	0	0	0	0	0	0	1	1	0	0	
GT44	0	0	0	0	0	0	1	0	0	0	0	
GT48	1	1	1	1	4	1	3	1	1	1	1	
GT49	0	0	0	0	0	0	0	0	0	0	0	
GT5	1	2	2	2	5	0	0	0	2	1	0	
GT50	1	1	1	2	1	1	1	1	1	2	1	
GT54	0	0	0	0	0	0	0	1	0	0	0	
GT55	0	0	0	0	0	0	0	0	1	0	0	
GT57	2	2	2	2	2	3	2	2	2	1	2	
GT58	1	1	1	1	1	1	1	1	1	0	1	
GT59	1	1	1	1	1	2	1	1	1	1	1	
GT60	0	0	0	0	0	0	0	0	0	0	1	
GT62	3	3	3	3	2	0	3	3	3	3	0	
GT64	0	0	0	0	0	0	0	2	0	0	0	
GT65	0	0	0	0	0	0	0	0	1	0	0	
GT66	1	1	1	1	1	1	1	1	1	1	1	
GT68	3	2	1	2	0	0	0	0	0	3	0	
GT69	0	0	0	0	0	0	0	2	2	2	3	
GT71	4	3	4	4	0	0	6	5	1	2	2	
GT76	1	1	1	1	1	0	1	1	1	1	1	
GT77	0	0	0	0	0	0	0	2	1	0	0	
GT78	0	0	0	0	0	0	0	0	0	0	0	
GT8	8	6	6	4	3	2	4	7	2	5	2	
GT83	0	1	0	0	0	0	0	0	1	0	0	
GT90	2	2	2	2	0	0	0	6	5	5	5	
GT92	0	0	0	1	0	0	0	0	0	1	0	
PL1	0	0	0	0	0	0	0	5	1	4	1	
PL12	0	0	0	0	0	0	0	0	0	0	1	
PL20	0	0	0	0	0	0	0	1	1	0	0	
PL22	0	0	0	0	0	0	0	0	0	0	1	
PL3	1	1	1	0	0	0	0	7	1	0	0	PCW (pectin)
PL4	0	0	0	0	0	0	0	3	1	0	0	
PL7	0	0	0	0	0	0	0	0	0	1	0	
PL9	0	0	0	0	0	0	0	1	0	0	0	

Td: *T. deformans* , Tw: *T. wiesneri* , Tf: *T. flavorubra* , Tp: *T. populina* ,
Sp: S. pombe, Pj: Pneumocystis jirovecii, Sc: S. cerevisiae, Fg: Fusarium graminearum, Nc: Neurospora crassa, Ss: Sclerotinia sclerotiorum, Um: Ustilago maydis.
CW: Cell wall; ESR, energy storage and recovery; PCW: Plant cell wall; PG, protein glycosylation; FCW: Fungal cell wall

Table S8. Numbers of Plant Hormone Biosynthesis related genes in *Taphrina* species

	Gene name	*T. wiesneri*	*T. deformans*	*T. flavorubra*	*T. populina*
Auxin related genes					
	Tam	Tw_0539800.1<br>Tw_0068000.1	Td_0045500.1<br>Td_0263800.1	Tf_0075800.1<br>Tf_0034800.1	Tp_0005600.1<br>Tp_0431100.1
	iad	Tw_0363900.1	Td_0302900.1	Tf_0371200.1	Tp_0310400.1
	YUC	Tw_0040600.1<br>Tw_0486000.1	Td_0494600.1<br>Td_0270800.1	Tf_0058600.1<br>Tf_0112000.1	Tp_0416900.1
Cytokinin related genes					
	tRNA-IPT	Tw_0394100.1	Td_0154500.1	Tf_0655100.1	Tp_0463300.1
	CYP736A	Tw_0071600.1	Td_0413200.1	Tf_0030900.1	Tp_0229800.1
	LOG	Tw_0594900.1	Td_0545400.1	Tf_0152400.1	Tp_0197700.1
Gibberellin related genes					
	GA2ox/GA3ox/GA21ox	Tw_0346000.1	Td_0543900.1	Tf_0290500.1	ND
	GA4 desaturase	Tw_0023800.1	Td_0577000.1	Tf_0276700.1	ND
Abscisic acid related genes					
	BcABA1, BcABA2	Tw_0034000.1<br>Tw_0630300.1<br>Tw_0337600.1	Td_0370700.1<br>Td_0583200.1<br>Td_0377100.1	Tf_0065800.1<br>Tf_0609200.1<br>Tf_0570500.1	Tp_0019300.1
	ABA4/SDR	Tw_0190200.1	Td_0219700.1	Tf_0597000.1	Tp_0507500.1
	NCED	Tw_0006100.1	Td_0481100.1	Tf_0327700.1	Tp_0175700.1
	ABAO	Tw_0206000.1	Td_0198000.1	Tf_0174800.1	Tp_0021900.1

ND; not detected

Table S9 – *Taphrina* genomic and RNAseq mapping statistics

Library type	species name	Total reads	Mapped reads	Duplicates*	Proper pairs	Both Pairs mapped	Singletons	Average Insert size (bp)
Genomic	*T. wiesneri*	66,770,014	97.36%	3.60%	95.79%	96.38%	0.98%	221
Genomic	*T. deformans*	39,535,106	98.06%	3.43%	94.11%	96.98%	1.09%	245
Genomic	*T. populina*	81,440,614	74.23%	15.99%	70.69%	71.96%	2.27%	227
Genomic	*T. flavorubra*	21,172,960	97.75%	2.11%	93.85%	96.55%	1.20%	324
RNAseq	*T. wiesneri*	14,380,398	96%	NA	83.69%	93.75%	2.44%	170
RNAseq	*T. deformans*	11,631,387	96%	NA	50.13%	93.31%	2.99%	180
RNAseq	*T. populina*	14,860,360	91%	NA	78.08%	87.58%	3.56%	167
RNAseq	*T. flavorubra*	3,978,619	20%	NA	16.28%	18.24%	1.47%	165

PCR duplicates were only marked in genomic DNA libraries
